# Identifying pathways to achieve diverse objectives of the National Essential Diagnostics List: developing an assessment framework based on field studies in Cambodia, Indonesia, Lao PDR, and the Philippines

**DOI:** 10.1186/s41182-025-00839-w

**Published:** 2025-11-06

**Authors:** Shogo Kanamori, Yuriko Egami, Eiichi Shimizu, Shinsuke Miyano, Antonio F. dela Resma Villanueva, Naofumi Hashimoto, Hiroyuki Kiyohara, Masataro Norizuki, Manami Uechi, Kyoko Koto-Shimada, Yasunori Ichimura, Masami Fujita

**Affiliations:** 1 Bureau of Global Health Cooperation, Japan Institute for Health Security, 1-21-1 Toyama, Shinjuku-ku, Tokyo, 162-8655 Japan; 2 Healthcare Unit, Economic Research Institute for ASEAN and East Asia, 6/f Sentral Senayan II, No. 8 Asia-Africa, Gelora Bung Karno, Senayan, Jakarta Pusat, 10270 Indonesia; 3 Independent Consultant, Flat 3, No. 4 Rose Street, Salama Park, Lusaka, Zambia

**Keywords:** Diagnostics, Low- and middle-income countries, Health systems, Health service delivery

## Abstract

**Background:**

Access to essential in vitro diagnostics is limited in many low- and middle-income countries (LMICs). To address this, the World Health Organization introduced the Model List of Essential In Vitro Diagnostics (EDL) in 2018, encouraging member states to develop National Essential Diagnostics Lists (NEDLs). To date, five LMICs—Burkina Faso, Ethiopia, India, Nepal, and Nigeria—have developed NEDLs. However, gaps remain in the objectives of NEDL development, which may undermine their effective use. In this study, we aimed to analyze the objectives of NEDL development through a literature review, elicit potential pathways for achieving these objectives, and develop an assessment framework for defining NEDL objectives and pathways.

**Methods:**

We analyzed the WHO EDL and NEDL documents and other relevant materials, aligning the objectives mentioned in these documents with a logic model. A provisional assessment framework was then designed and applied to examine stakeholder perspectives on NEDL objectives, which were obtained through key informant interviews in four Association of Southeast Asian Nations (ASEAN) countries—Cambodia, Indonesia, Lao PDR, and the Philippines—where NEDL initiatives are still in their early stages. Based on these findings, a revised assessment framework was developed.

**Results:**

The literature review yielded a provisional assessment framework comprising six domains: procurement, supply chain, laboratory equipment maintenance, quality assurance, regulatory work, and benefit packages. An analysis of stakeholder perspectives in the four ASEAN countries identified potential pathways for achieving the NEDL objectives across these six domains and other aspects. This process resulted in a new seven-domain assessment framework, incorporating key modifications to the provisional framework: “benefit packages” was replaced with “health financing”, and “service delivery platform” was added as the seventh domain.

**Conclusions:**

The newly developed assessment framework can support high-level officials in initiating NEDL development and promoting its effective use. It can also guide health ministries in selecting NEDL leads and technical committee members and help identify intermediary outcomes for monitoring NEDL operationalization. Although empirical evidence on NEDL outcomes remains limited owing to its early global implementation stage, this study provides valuable insights to support the future development and deployment of NEDLs and strengthen diagnostic systems in LMICs.

## Background

The availability of essential in vitro diagnostic (IVD) tests is a prerequisite to achieve Universal Health Coverage in low- and middle-income countries (LMICs). However, 47% of the world population has little to no access to diagnostics [1]. Limited access in LMICs is driven primarily by inadequate resources, including funding, qualified personnel, and infrastructural capacity [2–4]. Geographic disparities further restrict access in remote areas [4, 5]. Additional health system-related barriers include unstable supply chains, insufficient quality assurance systems, procurement challenges, complex registration and regulatory processes, and inadequate capacity for medical equipment maintenance [3, 4, 6–9]. On the demand side, patients often face high out-of-pocket expenditures and transportation costs, limited awareness and knowledge, concerns regarding service reliability, disease-related stigma, and discomfort or embarrassment associated with testing [5, 6, 10–12].

To address these challenges, the World Health Organization (WHO) launched the Model Essential In Vitro Diagnostics List (EDL) in 2018, which was updated in its fourth edition in 2023 [13]. The EDL, developed by the WHO Strategic Advisory Group of Experts on IVDs, provides evidence-based recommendations on essential IVDs and includes cost-effectiveness studies of the listed tests. It serves as a reference for countries to guide the development or revision of their national EDLs (NEDLs), as emphasized in the 76th World Health Assembly (WHA76.5). This resolution urged member states to adapt the WHO EDL to local contexts, identify and address diagnostic gaps, and regularly update these lists [14]. Further practical guidance, including the importance of political commitment and a committee-led process, was outlined in another WHO publication [15]. To date, five LMICs—Burkina Faso [16], Ethiopia [17], India [18], Nepal [19], and Nigeria [20]—have developed NEDLs.

In the Association of Southeast Asian Nations (ASEAN) Region, the WHO and the Economic Research Institute for ASEAN and East Asia (ERIA) initiated a gap analysis in 2023 on IVD availability in selected countries, followed by regional consultative meetings to promote NEDL development among ASEAN member states [21]. Building on these efforts, in May 2024, the ERIA and the Japan Institute for Health Security (JIHS), under the Ministry of Health, Labor and Welfare of Japan, launched the partnership project “Initial Assessment for the Development of National Essential Diagnostics List in ASEAN Countries”. Between December 2024 and May 2025, the JIHS and ERIA conducted an initial assessment of NEDL development in Cambodia, Indonesia, Lao PDR, and the Philippines, which are currently in the preparatory phase, to suggest approaches to maximize NEDL utility within each national context.

The overarching goal of the NEDL is to improve access to essential IVDs, whereas its immediate objective is to provide a priority IVD list [13]. However, information gaps remain regarding intermediary objectives that connect immediate objectives and goals. A previous study highlighted that decision-makers often lack a clear understanding of the purpose and significance of developing an NEDL [22]. Therefore, the concrete mechanisms or potential pathways through which NEDL development translates into improved access to essential IVDs are poorly understood. This lack of clarity regarding its objectives could undermine the effective utilization of NEDLs, as leaders must fully understand its objectives to effectively translate policy into practice [23]. Because the NEDL is relatively new, its empirical objectives have not been well documented. In contrast, several studies have reported lessons from the implementation of the Essential Medicines List (EML), which has been in place since 1977 [24]. Successful cases have shown that the EML improved access to essential medicines by enhancing procurement and supply chains [25]; lowering prices, thereby reducing out-of-pocket expenditures for patients [26]; and facilitating the registration of WHO-prequalified products [27]. These lessons provide valuable insights for identifying intermediary objectives in NEDL development, which can help high-level officials initiate and support the development process. Against this backdrop, in the present study, we aimed to (1) analyze the objectives of NEDL development through a literature review; (2) elicit stakeholders’ perceptions of potential pathways for achieving these objectives in four ASEAN countries; and (3) develop a new assessment framework to define NEDL objectives and pathways.

## Methods

### Literature review to analyze NEDL objectives and develop a provisional assessment framework

The study team, consisting of three authors with strong local contextual knowledge and experience in assessing NEDLs in LMICs, reviewed the WHO EDL [13] and NEDL publications from five countries, namely Burkina Faso [16], Ethiopia [17], India [18], Nepal [19], and Nigeria [20], to extract the NEDL development objectives that were explicitly articulated in these documents. The extracted objectives were aligned with the logic model, which is a systematic and visual framework that illustrates the perceived relationships between resources, activities, and results [28]. They were then arranged in a hierarchy that progressed from immediate objectives to ultimate goals, based on their causal linkages—from short-term or process-oriented results to long-term outcomes and impacts. To facilitate analysis, the team adapted this framework into four levels: Level 1 (immediate), Level 2 (intermediary), Level 3 (overarching), and Level 4 (ultimate). Additionally, the team reviewed research articles indexed in PubMed and the relevant gray literature that described the objectives of the NEDL. The team then discussed the findings and developed a provisional framework that comprehensively and logically captured the Level 2 (intermediary) objectives of NEDL development for subsequent assessment of NEDL objectives in the contexts of four ASEAN countries.

### Identifying potential pathways for achieving NEDL objectives in four ASEAN countries

The study team reviewed field mission reports from initial assessments conducted in Cambodia, Indonesia, Lao PDR, and the Philippines (unpublished data), along with relevant government guidelines and reports. A qualitative content analysis approach was applied to systematically review the mission reports. Statements and observations related to stakeholders’ views on NEDL development, implementation, challenges, and expected outcomes, which had been obtained through key informant interviews, were extracted and coded according to the components of the provisional assessment framework. The team analyzed the coded statements and observations to identify potential pathways in which the NEDL might contribute to achieving the Level 2 (intermediary) objectives in the contexts of the four countries.

### Developing a revised assessment framework

The study team discussed the findings and developed a new assessment framework to define the NEDL objectives and potential pathways to achieve them. After deliberating the need to revise the existing framework, the team reached a consensus on the modified version.

## Results

### Provisional assessment framework informed by the literature review

The WHO EDL and NEDL publications from Burkina Faso, Ethiopia, India, Nepal, and Nigeria outline the objectives for NEDL development at different levels, although no such objectives were stated in the documents from Burkina Faso and India. These objectives were presented in varying formats, either as paragraphs or bullet points, and spanned multiple levels. The study team interpreted the narratives and converted them into bullet points to standardize them. Subsequently, the team discussed their classification within a logic model and reached a consensus to categorize them according to their causal relationships (Table 1).

**Table 1:** Summary of objectives stated in the WHO EDL and NEDL publications of five countries

Publications	Objective levels based on causal relationships			
	Level 1 (immediate)	Level 2 (intermediary)	Level 3 (overarching)	Level 4 (ultimate)
WHO EDL [13]	Providing a priority list of IVDs to guide allocation of limited resources	Improving selection, procurement, supply, donation, and provision of IVDs	Improving access to essential IVDs	
NEDL Burkina Faso [16]	(Not stated)			
NEDL Ethiopia [17]	Providing a priority list of IVDs	Improving selection, procurement, supply, donation, and provision of IVDs	Improving access to essential health services	
NEDL India [18]	(Not stated)			
NEDL Nepal [19]			Improving access to essential IVDs	Reducing the antimicrobial resistance rates Identifying contagious diseases promptly
NEDL Nigeria [20]	Providing a priority list of IVDs	Improving selection, procurement, supply, donation, and provision of IVDs		

EDL: essential diagnostics list; NEDL: National Essential Diagnostics List; IVD: In vitro diagnostics

“Providing a priority list of IVDs” was designated as a Level 1 (immediate) objective (WHO, Ethiopia, and Nigeria), whereas “improving access to essential IVDs” was categorized as a Level 3 (overarching) objective (WHO, Ethiopia, and Nepal). In between, “improving selection, procurement, supply, donation, and provision of IVDs” was classified as a Level 2 (intermediary) objective—the primary focus of this study and the basis for developing a provisional assessment framework (WHO, Ethiopia, and Nigeria). Finally, “reducing the antimicrobial resistance rates” and “identifying contagious disease promptly” were classified as Level 4 (ultimate) objectives (Nepal), as they can only be achieved after attaining the Level 3 (overarching) objectives.

To further elaborate on the Level 2 (intermediary) objectives beyond “improving selection, procurement, supply, donation, and provision of IVDs”, the study team also reviewed research articles and gray literature. The review identified one research article [29] and conference materials [30] as sources to fill information gaps, which together indicated six intermediary objectives associated with procurement, supply chain, equipment maintenance, quality assurance, regulatory work, and benefits packages. These six domains were used to develop a provisional assessment framework that included key questions on how the NEDL could contribute to improving them (Table 2).

**Table 2:** Provisional framework for assessing NEDL objectives

Domains	Key questions
1. Procurement	How can NEDLs contribute to better procurement of IVDs?
2. Supply chain	How can NEDLs contribute to a better supply chain for IVDs?
3. Laboratory equipment maintenance	How can NEDLs contribute to better laboratory equipment maintenance?
4. Quality assurance	How can NEDLs contribute to better quality assurance of IVDs?
5. Regulatory work	How can NEDLs contribute to more efficient regulatory work for IVDs?
6. Benefit packages	How can NEDLs contribute to more streamlined benefit packages?

NEDL: National Essential Diagnostics List; IVD: in vitro diagnostics

### Pathways for achieving NEDL objectives in the four ASEAN countries

As documented in the field mission reports, the stakeholders interviewed across the four ASEAN countries included health ministry offices, various health facilities, provincial and district health authorities, health insurance agencies, and private-sector manufacturers. A consolidated list of interviewed stakeholders is presented in Table 3. An analysis of these reports summarizing stakeholders’ perceptions of potential pathways through which the NEDL could contribute to improvements across the six domains and other aspects based on the provisional assessment framework is outlined below.

**Table 3:** List of the interviewed stakeholders

	Cambodia	Indonesia	Lao PDR	Philippines
Health Ministry—Laboratory	X	X	X	X
Health Ministry—Regulatory	X	X	X	X
Health Ministry—Medical Store	X		X	
Health Ministry—NCD		X	X	X
Health Ministry—Communicable Disease	X	X	X	X
Health Ministry—Sub-national Office				X
Health Ministry—Other Offices	X	X	X	X
National Hospital	X	X	X	X
Regional/Provincial/District Hospital	X	X	X	X
Health Center	X	X	X	
National/Sub-national Reference Laboratory	X	X	X	X
Provincial/District Health Office	X	X	X	
Health Insurance Bureau	X	X	X	X
Private manufacturer	X			

NCD: noncommunicable disease

### Procurement of IVDs

*Serving as a guide for priority IVDs, enabling more focused procurement (Cambodia, Indonesia, Lao PDR, Philippines):* documented priority IVDs in the NEDL can guide procurement processes, particularly in resource-limited settings where not all essential IVDs can be made available. In Indonesia, following the issuance of the new public health laboratory standards in 2024 [31], the Ministry of Health has been strengthening laboratory capacities, and the NEDL could serve as a reference for priority procurement.

*Lowering IVD prices (Cambodia, Indonesia, Philippines): *the prioritization of IVDs may increase supplier registration and participation in competitive bidding, and potentially lower prices. Bulk procurement through central systems could further reduce costs.

*Facilitating decision-making for introducing new IVDs at health facilities (Indonesia, Philippines)*: the NEDL could guide prioritization when clinicians request multiple IVDs and justify introducing new tests to health facility management.

*Improving the efficiency of procurement officers’ work (Philippines):* the NEDL can serve as a reference for specifying the IVDs to be procured, especially when product information is incomplete or obsolete.

### Supply chain for IVDs

*Enabling better forecasting and procurement planning (Cambodia, Indonesia, Lao PDR, Philippines)*: standardized test requirements in the NEDL facilitate focused forecasting and procurement planning.

*Enabling budget allocation for priority IVDs (Indonesia)*: recognizing essential IVDs enables governments and hospitals to strategically allocate resources, ensuring a sufficient stock of priority tests.

*Promoting rigorous monitoring of IVD stocks (Indonesia)*: inclusion in the NEDL allows authorities to monitor availability more effectively, similar to the EML.

*Reducing bidding failures (Philippines):* streamlined procurement via the NEDL may decrease instances of bidding failure, which is a major cause of stockouts.

### Maintenance of laboratory equipment

*Improving training planning and implementation for equipment maintenance (Indonesia, Lao PDR, Philippines)*: the standardization of IVD equipment can enhance the planning and implementation of maintenance training for maintenance technicians assigned to hospitals and laboratories.

*Focusing suppliers on limited equipment types (Cambodia)*: standardization reduces the variety of equipment, allowing suppliers to concentrate resources without compromising diagnostic capacity.

### Quality assurance (QA) of IVDs

*Guiding priority tests when QA coverage expands (Cambodia, Indonesia, Lao PDR, Philippines)*: although the NEDL may not directly affect current external quality assurance (EQA) practices, it can serve as a reference when expanding the coverage of tests under EQA programs.

### Regulatory work for IVDs

*Promoting fast-track registration (Indonesia, Philippines)*: the NEDL, or its development process, may prompt the Health Ministry to adopt fast-track registration for listed IVDs.

*Guiding expansion of regulatory coverage (Philippines)*: the current regulatory framework in the Philippines requires only eight IVDs for mandatory registration [32]. The NEDL helps prioritize IVDs when the Department of Health expands the coverage of IVDs in its regulatory framework.

*Triggering the creation of an IVD category in the registration system (Cambodia)*: in Cambodia, IVDs are registered as part of the medical equipment, and there is no “IVD” category in the registration system. The NEDL may facilitate the establishment of a dedicated IVD category in the registration system, separate from general medical equipment.

### Benefit packages

*Improving benefit packages: *the NEDL could serve as a priority list of IVDs and as a reference *document* when benefit packages are updated.

*Facilitating cost analysis or health technology assessment (HTA) (Cambodia, Indonesia, Lao PDR)*: the NEDL could indicate priority IVDs for which cost analyses or HTAs should be conducted. In Cambodia, essential laboratory tests are intended to be covered by the national health insurance scheme. However, some tests (e.g., HbA1c) are so costly that health facilities cannot afford to provide them in outpatient services because their operating costs exceed case-based reimbursement. The introduction of the NEDL, or its development process, may trigger discussions on the cost analysis and insurance coverage of essential but expensive laboratory tests.

*Strengthening the coverage of laboratory tests in the Primary Care Package (Philippines):* in the Philippines, healthcare providers are required to deliver 13 laboratory and diagnostic services under the Primary Care Package, 11 of which are IVDs [33]. During future updates, the NEDL may enable the Philippine Health Insurance Corporation to expand or strengthen the coverage of laboratory tests included in the Primary Care Package.

### Other aspects

*Enabling better training planning for laboratory technologists and health workers on IVDs (Cambodia, Indonesia, Lao PDR, Philippines)*: by defining the priority IVDs to be made available at each level, the NEDL could facilitate more effective training plans for laboratory technologists and health workers.

*Complementing the existing guidance by disease control programs (Cambodia, Indonesia, Lao PDR, Philippines)*: disease control programs, such as tuberculosis (TB), HIV/AIDS, and malaria programs, provide guidelines or manuals indicating essential IVDs; however, they may not necessarily be comprehensive or exhaustive. Through the Technical Working Group, the NEDL development process could serve as a forum for discussions with disease control programs on additional essential IVDs that are not listed in their manuals. Importantly, this could help integrate siloed systems created by disease control programs and broaden their scope to address other diseases.

*Facilitating the update of accreditation criteria for hospitals and public health laboratories (Indonesia)*: the NEDL could serve as a reference list for IVDs when the Ministry of Health updates the accreditation criteria for hospitals and public health laboratories. In particular, the Ministry of Health is currently revising accreditation standards for public health laboratories Tiers 2 to 5 following the introduction of new public health laboratory standards in 2024 [31], during which the NEDL may serve as a key reference document.

*Guiding the establishment of supervision systems for clinical laboratories (Lao PDR)*: in Lao PDR, licensing systems exist for hospitals and health workers, but not for laboratories. Moreover, the Ministry of Health lacks mechanisms for inspecting laboratory practices in private hospitals. The NEDL, or its development process, could trigger discussions on the creation of such supervision systems.

*Strengthening the monitoring of noncommunicable disease (NCD) tests (Philippines)*: in the Philippines, there is no monitoring mechanism for NCD-related laboratory tests. The NEDL can guide the Department of Health in developing an information system for NCD tests, thereby enabling better monitoring.

*Facilitating inspections of laboratories and IVDs (Philippines)*: in the Philippines, the regional Department of Health offices periodically inspect clinical laboratories to ensure compliance with licensing standards [34]. They also review laboratory tests outside licensing requirements if those tests are performed. The NEDL could provide guidance for these inspections, particularly regarding tests that are not formally covered by licensing standards.

*Broadening the categories of health workers authorized to conduct diagnostic testing (Philippines)*: in the Philippines, official orders restrict laboratory tests, including point-of-care tests (POCTs), to medical laboratory technologists [34, 35]. However, in practice, health workers, such as nurses and midwives, perform some POCTs (e.g., glucose tests with a glucose meter) in primary facilities without laboratories. The NEDL development process highlights this discrepancy and encourages the Department of Health to develop new POCT policies at the primary level.

### New assessment framework for defining the NEDL objectives

Based on the findings from the four ASEAN countries, a consensus was reached on the new framework presented in Table 4, which comprises seven domains: procurement, supply chain, laboratory equipment maintenance, quality assurance, regulatory work, health financing, and service delivery platforms. Each domain is accompanied by potential pathways.

**Table 4:** New assessment framework for NEDL objectives

Domains	Potential pathways
Procurement	More focused procurement of IVDs Lowering IVD prices Facilitating decision-making for introducing new IVDs Helping procurement officers
Supply chain	Better forecasting of IVD requirements Better budget allocation More rigorous stock monitoring Less frequent bidding failure
Laboratory equipment maintenance	Better training planning for IVD maintenance Making suppliers focused on priority IVDs
Quality assurance	Guiding QA coverage expansion
Regulatory work	Facilitating fast-track registration of IVDs Guiding the regulatory coverage expansion of IVDs Creating the IVD category in the registration system
Health financing	Improving benefit packages Better cost analysis of IVDs Strengthening laboratory test coverage in primary care
Service delivery platforms	Better training planning for laboratory personnel Qualifying non-MLTs to perform POCTs Better oversight of laboratory services Complementing disease control programs’ guidance

IVD: in vitro diagnostics; QA: quality assurance; MLT: medical laboratory technologist

A key modification from the provisional assessment framework was the replacement of “benefit packages” with “health financing” in the sixth domain, as the latter was deemed more appropriate given its broader scope. In addition, “service delivery platforms” were added as a seventh domain to capture stakeholders’ perspectives on “other aspects”, and the perceived pathways to achieve objectives for each domain were summarized as potential pathways.

## Discussion

Analysis of the WHO EDL and existing NEDL publications in five countries indicates that the immediate objective of the NEDL is to provide a prioritized list of IVDs, while its overarching goal is to improve access to essential IVDs. However, the intermediary objectives that connect these two levels remain insufficiently defined. In this study, we identified multiple intermediary objectives through an additional literature review and revealed potential pathways to achieve them via stakeholder interviews in four ASEAN countries. Findings from the field studies further informed the development of a new assessment framework to define NEDL objectives and potential pathways. The framework comprises seven domains: procurement, supply chain, equipment maintenance, regulatory work, quality assurance, health financing, and service delivery platforms. It was developed based on key informant interviews with a comprehensive set of stakeholders, representing potential future users of NEDLs, thereby demonstrating its thoroughness and contextual relevance to the four ASEAN countries. Overall, the results suggest that the NEDL has the potential not only to guide the prioritization of IVDs, but also to act as a catalyst for broader health system strengthening, particularly across the seven domains.

The findings of this study could contribute to the future development and operationalization of NEDLs at the country level. First, the new assessment framework serves as a basis for defining NEDL objectives, thereby guiding governments in initiating the development process and using it effectively. Diagnostics play a crucial and foundational role in quality healthcare; however, their importance is often overlooked [1]. To prompt decision-makers to prioritize diagnostics, they must be adequately informed about feasible approaches. Presenting the assumptive objectives of the NEDL, along with potential pathways illustrating benefits at the county level, may help engage high-level officials in both the development and utilization stages of the NEDL.

Second, our findings provide useful insights into the NEDL development process itself. The WHO recommends that health ministries appoint a technical lead and committee, including a broad range of officials and specialists, to oversee NEDL development [15]. Experiences from India and Nigeria further highlight that stakeholder engagement is critical for institutionalizing NEDL [22, 29]. Local contexts and potential NEDL users should be considered when selecting technical leads and committee members. This study suggests that the potential users can span seven domains. These actors could either be included directly in the committee or engage through public consultations on the draft NEDL versions, thereby strengthening the development process and enhancing the realization of NEDL objectives.

Third, the findings may inform the creation of a monitoring framework at the operationalization stage of the NEDL. While the ultimate goal of the NEDL is to increase access to essential IVDs [15], guidance on monitoring progress toward this goal remains limited. The new framework, particularly the seven domains and potential pathways, may serve to define the intermediary outcomes that can be tracked as part of operational monitoring.

All seven domains are important intermediary objectives for achieving the overarching goal of improving access to quality IVDs. Among them, procurement and supply chains are directly linked to IVD availability. The study highlighted that “more focused procurement of IVDs” under the procurement domain and “better forecasting of IVD requirements” under the supply chain domain were consistently perceived as NEDL objectives across all four countries. These domains were also identified as primary objectives of the NEDL in India and Nigeria [20, 29], suggesting their broader relevance to other LMICs.

The provisional framework, which is based on the six objectives extracted from the literature review, was further refined through field studies in four ASEAN countries. Specifically, a seventh domain, “service delivery platforms”, along with potential pathways, was added, enhancing the framework’s comprehensiveness and alignment with these countries’ local contexts. This framework can be applied to analyze the intermediary objectives of NEDL development and potential pathways to achieve them in other countries; however, to further enhance its validity, additional field studies in diverse contexts are needed.

This study has some limitations. Because all four countries are still developing their NEDLs, empirical evidence of their impacts or benefits could not be obtained. Consequently, an analysis of objective settings largely relies on stakeholder perceptions. In addition, only one province outside the capital region was visited in each country, which may limit the representativeness of the stakeholder views. Moreover, studies on the antecedents of NEDL have been limited, as only five countries have developed one to date, constraining the available evidence for discussion in this paper.

## Conclusions

This study suggests that NEDLs can act as a catalyst for the broader health system strengthening, particularly in procurement, supply chains, equipment maintenance, regulatory work, quality assurance, health financing, and service delivery platforms. Furthermore, it resulted in the development of a new assessment framework for NEDL objective settings. The findings, including this framework, can help engage high-level officials in the development and effective utilization of the NEDL. They can also guide ministries in selecting technical leads and committee members and provide a basis for identifying intermediary outcomes for monitoring NEDL operationalization. Although empirical evidence on NEDL outcomes remains limited, given its early global stages, this study provides critical insights to support the future development and operationalization of NEDLs. This study also highlights the role of NEDLs in strengthening diagnostic systems in LMICs.

## Data Availability

No datasets were generated or analysed during the current study.

## Abbreviations

ASEAN: Association of Southeast Asian Nations
EDL: Essential in vitro diagnostics
EML: Essential medicines list
EQA: External quality assurance
ERIA: Economic Research Institute for ASEAN and East Asia
HTA: Health technology assessment
IVD: In vitro diagnostics
JIHS: Japan Institute for Health Security
LMIC: Low- and middle-income country
NCD: Noncommunicable disease
NEDL: National Essential Diagnostics List
POCT: Point-of-care test
QA: Quality assurance
WHO: World Health Organization
